# Testing for Spatial Neglect with Line Bisection and Target Cancellation: Are Both Tasks Really Unrelated?

**DOI:** 10.1371/journal.pone.0023017

**Published:** 2011-07-28

**Authors:** Pascal Molenberghs, Martin V. Sale

**Affiliations:** 1 School of Psychology, The University of Queensland, Brisbane, Queensland, Australia; 2 Queensland Brain Institute, The University of Queensland, Brisbane, Queensland, Australia; French National Centre for Scientific Research, France

## Abstract

Damage to the parietal lobe can induce a condition known as spatial neglect, characterized by a lack of awareness of the personal and/or extrapersonal space opposite the damaged brain region. Spatial neglect is commonly assessed clinically using either the line bisection or the target cancellation task. However, it is unclear whether poor performance on each of these two tasks is associated with the same or different lesion locations. To date, methodological limitations and differences have prevented a definitive link between task performance and lesion location to be made. Here we report findings from a voxel-based lesion symptom mapping (VLSM) analysis of an unbiased selection of 44 patients with a recent unifocal stroke. Patients performed both the line bisection and target cancellation task. For each of the two tasks a continuous score was incorporated into the VLSM analysis. Both tasks correlated highly with each other (r = .76) and VLSM analyses indicated that the angular gyrus was the critical lesion site for both tasks. The results suggest that both tasks probe the same underlying cortical deficits and although the cancellation task was more sensitive than the line bisection task, both can be used in a clinical setting to test for spatial neglect.

## Introduction

Visual neglect is defined as the inability to detect, attend or respond to stimuli in spatial locations contralateral to the side of cerebral damage [1]. The two tasks most commonly used to test for neglect in a clinical setting are the cancellation task [2] and the line bisection task [3]. It is currently unclear, however, whether the same underlying cortical processes are activated with these two tests for neglect. The line bisection and target cancellation task have been found to load on different factors in some studies [4] but others [5] found that different neglect tasks (including the line bisection and target cancellation task) all loaded high on the same factor. Patients with deficits on the line bisection task but not on the cancellation task (and vice versa) have been reported [6], [7], but overall patient performance on both tasks seems to be correlated [7]. Recently there has been some debate on the location of the critical lesion site for neglect. Some authors argue for the angular gyrus [8] while others [9], [10], [11] attribute this role to the superior temporal gyrus. One explanation for this discrepancy has been the use of different neglect tasks in these studies [12], [13]. Rorden et al. (2006) found that patients who have problems on the line bisection task have more posterior lesions (temporo-occipital junction) than patients who have problems on the target cancellation task. These latter patients have lesions in the superior temporal gyrus. In a recent study, Verdon et al. (2010) found that lesions in the right inferior parietal lobule were more associated with problems on the line bisection task, and lesions in the right dorsolateral prefrontal cortex were more associated with problems on the target cancellation task. Others only found a behavioral, but not an anatomical, separation between the two tasks [14]. If both tasks are uncorrelated, and test for different underlying brain lesions, this would have important implications for the use of these tasks in the everyday clinical setting. Therefore, the present study sought to resolve the controversy surrounding task performance and lesion location using an unbiased sample of 44 stroke patients. Rather than pre-categorizing the patients into dichotomous groups with an all-or-none approach to behavior, as in traditional subtraction and overlap approaches [12], [8], [9], a continuous measure was used in this VLSM analysis [15], [16]. This analysis method is the most appropriate for addressing the issue of task performance and lesion location as it utilizes continuous lesion location and behavioral data.

## Materials and Methods

All participants gave written informed consent in accordance with the Declaration of Helsinki. The ethical commission at the University Hospital Leuven approved the experimental protocol.

### Participants

**Figure 1:**
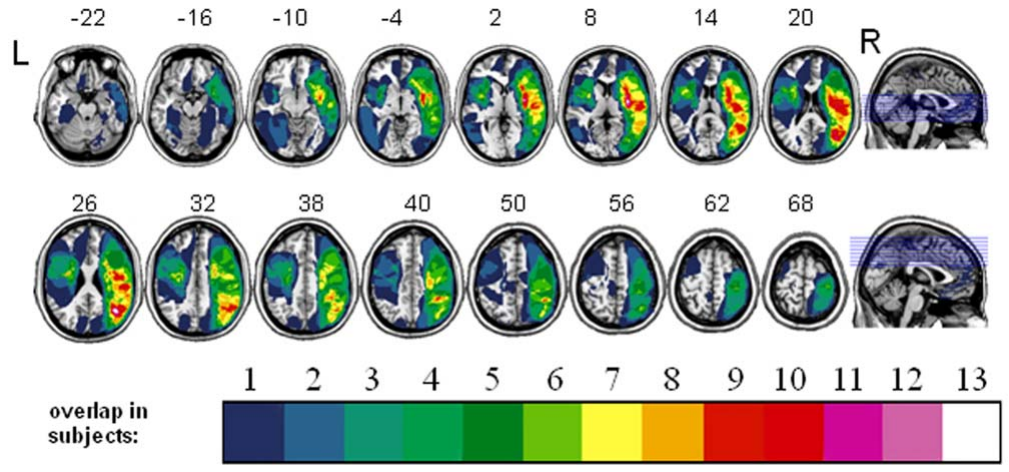
Lesion distribution volume map for all subjects (n = 44). The color code indicates in how many individuals a given voxel was lesioned (ranging from 1 to 13).

A consecutive series of 44 ischemic hemispheric stroke patients (See Table 1 for details) who had suffered a non-lacunar unifocal ischemic hemispheric stroke, confirmed on clinical Fluid Attenuation Inversion Recovery (FLAIR) or Diffusion-Weighted Imaging (DWI) magnetic resonance imaging (MRI) participated in the study. Participants were excluded if they were aged over 85 years, had a pre-existing periventricular or subcortical white matter lesions or a pre-existing stroke on MRI, had insufficient balance to sit independently, and general inability to understand and carry out the task. Although spatial neglect is more frequent after a right hemisphere lesion, it is not uncommon for patients to experience neglect also after a left hemisphere lesion [17]. Therefore both left- and right-sided patients were included in this study. The anatomical distribution of the ischemic lesions is shown in Figure 1. Visual fields were intact except in case 10 (left hemianopia), 14 (right lower quadrantanopia), 15 (left upper quadrantanopia) and 32 (left lower quadrantanopia).

**Table 1:** Behavioral parameters of the 44 stroke patients.

case	age	*lesion side*	*lesion size cm* ^^3^^	*days since stroke onset*	Bells omissions L_M_R	Line Bisection %	case	Age	*lesion side*	*lesion size cm* ^^3^^	*days since stroke onset*	Bells omissions L_M_R	Line Bisection %
**1**	43	R	26.9	4	0_0_1	+5.8	23	37	L	11.2	21	0_0_0	+0.4
2	82	R	20.2	5	1_0_0	+3.7	24	76	L	4.0	5	1_1_2	+4.6
3	44	R	302.7	4	1_0_1	−5.3	25	79	R	40.8	14	2_1_1	−3.8
4	69	L	19.0	6	0_0_0	+0.7	26	65	R	49.5	10	1_1_1	+4.0
5	53	L	108.0	4	3_0_3	+4.1	27	62	R	89.7	4	2_0_0	+5.3
6	88	R	84.1	7	2_2_4	+8.1	28	37	R	84.8	14	2_0_1	+0.9
7	72	L	46.8	3	2_4_0	−1.7	**29**	**42**	**R**	**43.4**	**6**	**4_3_1**	**+18.7**
8	65	R	17.0	5	2_0_0	+5.8	30	54	R	30.2	5	2_0_0	+6.6
9	80	R	20.8	6	0_0_0	+1.2	31	42	L	13.8	133	2_1_1	+2.1
**10**	**74**	**R**	**173.0**	**6**	**14_0_1**	**+20.5**	32	64	R	197.0	196	2_0_0	−5.3
11	73	L	16.4	4	0_0_0	−0.3	33	77	L	17.2	126	0_0_1	−5.9
12	79	L	4.8	3	2_1_1	+1.9	34	34	L	64.9	168	0_1_0	+3.9
13	79	L	2.1	6	0_0_1	+1.7	35	66	L	95.1	126	1_1_2	+0.5
14	47	L	13.9	5	0_1_0	+3.8	36	55	R	2.6	140	1_0_0	−3.6
15	52	R	14.3	147	2_1_0	−5.9	37	64	R	107.0	196	3_0_1	+4.1
16	68	R	11.0	154	0_0_0	−3.0	38	61	L	18.5	7	0_0_1	+5.2
**17**	**64**	**R**	**216.0**	**5**	**15_4_2**	**+18.4**	39	62	L	17.0	133	0_0_0	+0.4
**18**	**79**	**R**	**191.0**	**4**	**15_4_0**	**+33.4**	40	35	L	64.4	63	0_0_0	+0.1
19	75	R	15.4	3	2_1_0	+1.2	41	60	R	29.6	168	1_1_0	−1.8
20	74	R	117.0	7	0_0_1	−1.7	42	44	R	161.0	91	0_0_0	+5.0
**21**	**84**	**L**	**12.5**	**6**	**0_0_0**	**+9.6**	43	71	L	25.8	14	1_0_1	+2.1
22	61	L	1.0	217	0_0_0	+2.0	**44**	**80**	**R**	**64.6**	**126**	**3_1_0**	**+6.3**

Legend: L: Left. R: Right. M: Middle. Line Bisection: Mean percentage deviation. Positive values are deviations to the patient ipsilesional side. Patients that meet the criteria for spatial neglect are indicated in bold.

### Neuropsychological protocol

Participants completed two standard neuropsychological tests of neglect.

### Neglect task 1

The first task was the bells target cancellation task [2]. This task consists of seven columns presented on an A4 sheet of paper, each containing five targets (bells) and 40 distractors. Three of the seven columns are on the left side of the A4 sheet (15 targets), one is in the middle and three are on the right (15 targets). Participants were asked to cross out all the bells. The number of omissions on the contralesional side minus the number of omissions on the ipsilesional side was calculated, and used as a score in the VLSM analysis. Participants were classified as having spatial neglect if they had three additional omissions on the ipsilesional side compared to the contralesional side [2].

### Neglect task 2

The second test of neglect was the line bisection task [3]. Participants were required to bisect a number of lines (20) in half with varying lengths (100, 120, 140, 150 160, 180 and 200 mm) by placing a small pencil mark trough each line as close to the center as possible. The mean percentage deviation from the middle to the ipsilesional side over all the lines was used as a score in the VLSM analysis. Ipsilesional deviation above 9.5 percent was taken as an indicator of spatial neglect. This number corresponds to a value above the 99 percent confidence interval in a control group [3].

### Image acquisition and preprocessing

Each of the 44 patients had an MRI scan (see [18], [19] for a similar procedure in the same patients) with a 3 T Philips Intera system (Best, Netherlands) equipped with a head volume coil that provided T1 images (TR = 1975 ms, TE = 30 ms, in-plane resolution 1mm) as well as Fluid Attenuation Inversion Recovery (FLAIR) 3D images (TR = 10,741 ms, TE = 150 ms). Using SPM2 (http://www.fil.ion.ucl.ac.uk, Welcome Trust Centre for Neuroimaging, London, UK) the T1 and FLAIR images were co-registered. The T1 scan was normalized to the Montreal Neurological Institute (MNI) T1 template in Talairach space [20], [21]. The spatial normalization involved both linear (12 affine transformations) and nonlinear (7×9×7 basis functions, 16 reiterations) transformations [22]. High regularization was used to constrain the non-linear part of the algorithm and penalize unlikely deformations associated with the presence of lesions [22], [23]. The same normalization matrix was applied to the FLAIR images. The match between each patient's normalized brain and the brain template was carefully evaluated through visual inspection and use of a cross-hair yoked between the template image and the normalized image. After verification of the normalization, lesions were semi-automatically delineated using MRIcro version 1.37 (http://www.sph.sc.edu/comd/rorden/mricro.html) and intensity thresholds were set manually [16]. The lesion volumes were subsequently imported into the MRIcron lesion-symptom mapping software (http://www.sph.sc.edu/comd/rorden/mricron). A voxel was included in the analysis only if it was lesioned in at least 4 of the subjects. Each of the 2 parameters were entered separately into a VLSM analysis [16] that examined which of the voxels, when lesioned, were associated with significantly worse scores compared to patients in whom these voxels were intact (Brunner and Munzel t test [24]). The significance threshold was set at P<0.01, with a FDR correction for the brain search volume [16]. If this threshold didn't reveal a significant result, the threshold was lowered to P<0.05, with a FDR correction for the brain search volume [16]. Anatomical localization was carried out by visual comparison of the MRI-projected sections with corresponding slices from the Duvernoy brain atlas [25].

## Results

### Behavioral Data

The behavioral data are listed in Table 1. Of the 44 patients included, six patients met the stringent criteria for spatial neglect described earlier. These patients are highlighted in bold in Table 1. Across all patients, a one-way pearson correlation found that performance on the neglect task 1 correlated significantly (r = .76; p<0.001) with performance on the neglect task 2 score. Even if we restricted our analysis to the six patients that met our stringent criteria for spatial neglect we found a significant correlation between the two tasks (r = .79; p<0.03).

### VLSM analysis

**Figure 2:**
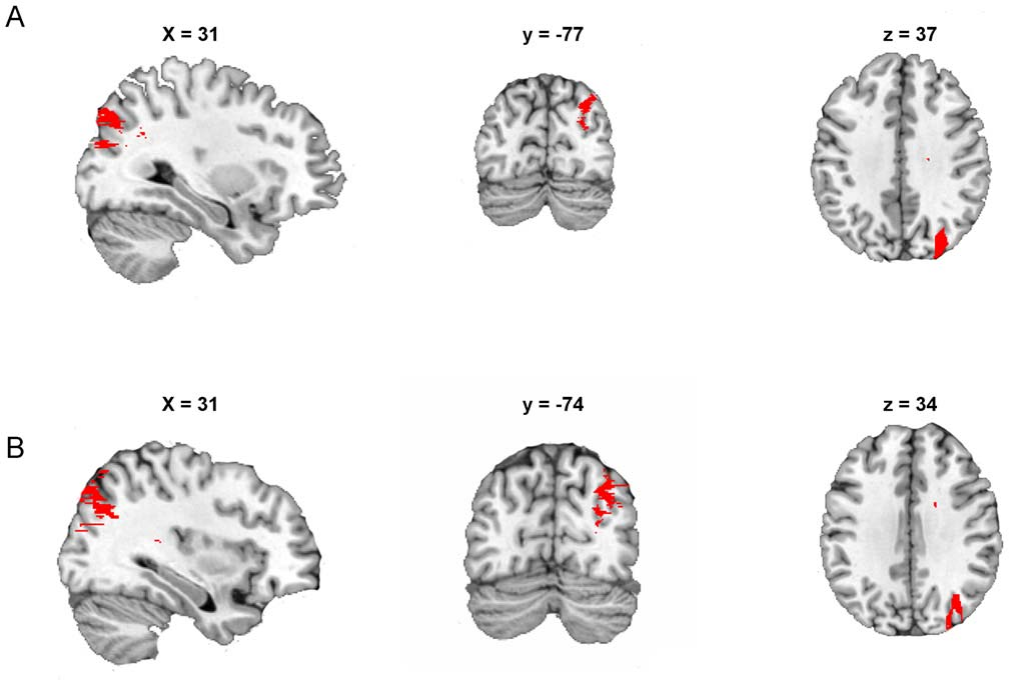
Lesion-symptom maps for all subjects (n = 44) showing results for the target cancellation task (neglect score 1, A), and the line bisection task (neglect score 2, B). With both tasks, the posterior medial part of the angular gyrus (red areas) was associated with significant deficits. The input threshold for the cancellation task was set at FDR<0.01, and for the line bisection task it was set at FDR <0.05.

The VLSM analysis revealed that poor performance on the neglect task 1 (cancellation task), and thus significantly more contralesional minus ipsilesional errors, was associated with a lesion of the posterior medial part of the right angular gyrus (MNI coordinate center of mass: x = 31, y = -77, z = 37, ext. 2048 mm^3^, P*<*0.01) compared with patients in whom this region was intact (Fig. 2A).

Using the initial significance threshold (FDR P<0.01), no significant voxels were revealed with the VLSM analysis for the neglect task 2 score (line bisection task). However, when the threshold was lowered to FDR P<0.05, lesions of the posterior medial part of the right angular gyrus (center of mass: x = 34, y = -74, z = 34, ext. 3723 mm^3^, P*<*0.05) were associated with significantly more ipsilesional deviation on the line bisection task than was seen in patients in whom this region was intact (Fig. 2B).

## Discussion

The principle result from the present study is that stroke patients with a lesion to the angular gyrus have impairments with both the line bisection and cancellation task, the two most commonly used clinical tests for spatial neglect. The relationship between lesion location and outcomes of clinical tests for spatial neglect have been controversial. This result is in agreement with some studies that suggest that deficits in scores for the two tasks are due to the same lesion site [14], [18], but is contrary to others [12], [13]. Unlike previous studies investigating the link between lesion location and task performance, the findings of the present study were obtained using VLSM analysis, which is the most appropriate tool for analyzing the link between lesion location and clinical scores. This is because VLSM does not require patients to be assigned into groups based on lesion location or behavioral score, but utilizes continuous lesion and behavioral data.

It is important to note that in this study an unbiased sample of stroke patients was used with a large variation in lesion site and clinical symptoms. Fourteen percent (6 out of 44) of the unbiased stroke patients tested met our stringent criteria for spatial neglect. Five of these patients had a right hemisphere lesion and one patient had a left hemisphere lesion. These values correspond with earlier results [17] that report less frequent occurrence of egocentric spatial neglect in left hemisphere damaged stroke patients compared to right hemisphere damaged stroke patients. The relative low number of neglect patients identified in the present study from the cohort of patients selected can be attributed to the stringent criteria we used to classify neglect patients compared to those often used in other studies (e.g. 9.5% ipsilesional deviation and three additional omissions on the contralesional side compared to the ipsilesional side was taken as a criterion for spatial neglect rather than three omissions overall). It is important to note, however, that spatial neglect is not an all-or-nothing disorder and that it is better represented on a continuous scale. Therefore contrary to other MRI lesion mapping studies with neglect patients [8], [9], patient lesions in this study were not dichotomously subdivided *a priori* into neglect or non-neglect patients. In a continuous VLSM-analysis the lesion of a patient who has a high score on the neglect factor counts more than a patient with a lower score [13], [15], [16], [26], [27], [28]. This procedure is tolerant of larger data variability and therefore produces more accurate lesion maps.

The result of the present study strengthens the view that both tasks are valid tools to test for spatial neglect in a clinical setting in a typical variable patient group. The finding of a critical lesion site in both tasks corresponds with earlier studies that show that the right parietal lobule [8], [18], [29], [30], [31] rather than the right superior temporal gyrus [9], [10] is associated with spatial neglect. Some studies suggest that neglect doesn't always result from a specific lesion site but can be a result of a disconnection in white matter pathways connecting parietal and frontal areas [32], [33], [34], [35], [36], [37], [38]. This is an important insight but it must be noted that most of the lesion damage in neglect patients is often situated in the gray matter structures rather than lesions to perisylvian white matter fiber tracts [39]. Therefore identifying the critical lesion site in neglect patients is still important for a better understanding of the anatomical basis of spatial neglect.

The VLSM result of the present study is in line with previous studies [12], [40] that suggest that the target cancellation (significant result at FDR 0.01) task is a more sensitive test for neglect than the line bisection task (significant result at FDR 0.05). Nevertheless, overall the results suggest that both tasks are valid tools to test for spatial neglect in a clinical setting as long as the tasks are used appropriately (e.g. stroke patients tested for spatial neglect in a clinical setting are often presented with a single horizontal line on a sheet of paper, but it is important to use multiple lines to get a valid result on the line bisection task [3]).

To conclude, the results of the present study suggest that spatial neglect is a disorder usually associated with right parietal damage to the angular gyrus. The deficits associated with spatial neglect can be tested in the clinical setting with both the target cancellation and line bisection tasks.
